# Functional characterization of the DUF1127-containing small protein YjiS of *Salmonella* Typhimurium

**DOI:** 10.1093/femsml/uqae026

**Published:** 2025-01-03

**Authors:** Elisa Venturini, Sandra Maaß, Thorsten Bischler, Dörte Becher, Jörg Vogel, Alexander J Westermann

**Affiliations:** Institute of Molecular Infection Biology (IMIB), University of Würzburg, D-97080 Würzburg, Germany; Institute of Microbiology, Department of Microbial Proteomics, University of Greifswald, D-17489 Greifswald, Germany; Core Unit Systems Medicine, University of Würzburg, D-97080 Würzburg, Germany; Institute of Microbiology, Department of Microbial Proteomics, University of Greifswald, D-17489 Greifswald, Germany; Institute of Molecular Infection Biology (IMIB), University of Würzburg, D-97080 Würzburg, Germany; Helmholtz Institute for RNA-based Infection Research (HIRI), Helmholtz Centre for Infection Research (HZI), D-97080 Würzburg, Germany; Helmholtz Institute for RNA-based Infection Research (HIRI), Helmholtz Centre for Infection Research (HZI), D-97080 Würzburg, Germany; Department of Microbiology, Biocenter, University of Würzburg, D-97074 Würzburg, Germany

**Keywords:** YjiS, small protein, *Salmonella* Typhimurium, DUF1127, dual RNA-seq, co-immunoprecipitation, macrophage, host–pathogen interaction, mass spectrometry, flagella, SsrA/B

## Abstract

Bacterial small proteins impact diverse physiological processes, however, technical challenges posed by small size hampered their systematic identification and biochemical characterization. In our quest to uncover small proteins relevant for *Salmonella* pathogenicity, we previously identified YjiS, a 54 amino acid protein, which is strongly induced during this pathogen’s intracellular infection stage. Here, we set out to further characterize the role of YjiS. Cell culture infection assays with *Salmonella* mutants lacking or overexpressing YjiS suggested this small protein to delay bacterial escape from macrophages. Mutant scanning of the protein’s conserved, arginine-rich DUF1127 domain excluded a major effect of single amino acid substitutions on the infection phenotype. A comparative dual RNA-seq assay uncovered the molecular footprint of YjiS in the macrophage response to infection, with host effects related to oxidative stress and the cell cortex. Bacterial cell fractionation experiments demonstrated YjiS to associate with the inner membrane and proteins interacting with YjiS in pull-down experiments were enriched for inner membrane processes. Among the YjiS interactors was the two-component system SsrA/B, the master transcriptional activator of intracellular virulence genes and a suppressor of flagellar genes. Indeed, in the absence of YjiS, we observed elevated expression of motility genes and an increased number of flagella per bacterium. Together, our study points to a role for *Salmonella* YjiS as a membrane-associated timer of pathogen dissemination.

## Teaser

Alphofold structure of the *Salmonella* small protein YjiS and electron microscopy visualization of flagella of a *yjiS* deletion mutant.

## Introduction

Small bacterial proteins (loosely defined as <100 amino acids [aa] in size) represent a class of understudied macromolecules that only recently entered the scientific spotlight (Orr et al. 2020). Small protein-coding genes are challenging to map in bacterial genomes, due to difficulties in distinguishing true short open reading frames (sORFs) from untranslated sequences (Poptsova and Gogarten 2010). In addition, the functional characterization of small proteins comes with its own challenges. For example, tagging small proteins with epitopes bears the risk of disrupting proper folding, affecting sub-cellular localization, or blunting their interaction with cellular ligands. These difficulties notwithstanding, there is an increasing number of studies that successfully identified (Miravet-Verde et al. 2019, Weaver et al. 2019) and functionally characterized (Wilmaerts et al. 2019, Yin et al. 2019, Hör et al. 2020, Yadavalli et al. 2020, Choi et al. 2021) small proteins in diverse bacterial species. This list includes a recent comprehensive study in *Salmonella enterica* serovar Typhimurium (henceforth, *Salmonella*) wherein the integration of several different high-throughput data sets (gene expression, ribosome profiling, mutant fitness data) and *in silico* predictions enabled the annotation of a total of 609 small proteins and candidates thereof in this species (Venturini et al. 2020).

Considering that *Salmonella* might be the best-characterized bacterial pathogen to date, it lends itself as a model species for the discovery of small protein functions in bacterial virulence. *Salmonella* virulence factors are well defined and are largely encoded on two horizontally acquired *Salmonella* pathogenicity islands (SPIs), referred to as SPI-1 and SPI-2 (Patel and Galán 2005, Jennings et al. 2017). Both these gene clusters encode a type-III secretion system and the correspondingly secreted effector proteins, which are typically themselves small in size. SPI-1 effectors are responsible for invading non-phagocytic host cells and SPI-2 effectors are required to create an intracellular environment that is favorable for *Salmonella* survival. This lifestyle switch is tightly regulated at both the transcriptional (Pérez-Morales et al. 2017) and post-transcriptional level (Westermann et al. 2016), underscoring the importance of appropriate expression of distinct virulence programs along the infection cycle.

**Figure 1.**
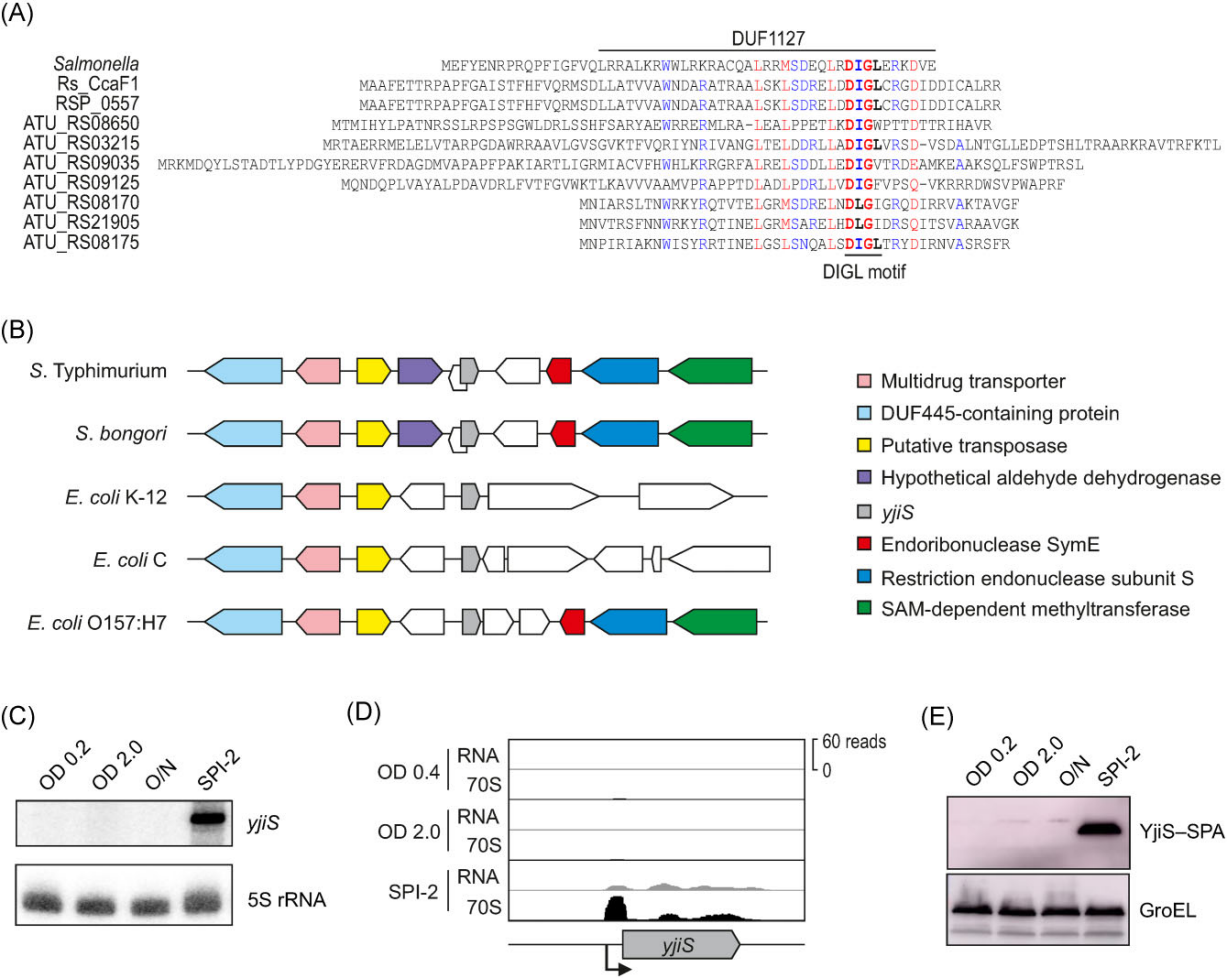
Prevalence of DUF1127-containing small proteins in Proteobacteria and expression of *Salmonella* YjiS. (A) Alignment of the amino acid sequences of DUF1127-containing small proteins from *Salmonella enterica* serovar Typhimurium strain SL1344, *Rhodobacter sphaeroides* (Rs_CcaF1 and RSP_0557), and *Agrobacterium tumefaciens* (ATU). Amino acids are colored based on their conservation: black, not conserved; blue, poorly conserved (50–90% consensus); red, highly conserved (>90% consensus). (B) Schematic representation of the *yjiS* genomic locus in select *Salmonella* and *Escherichia* species/strains. Protein classes conserved among different species are colored as shown in the legend. The synteny analysis was performed and visualized using SyntTax (Oberto 2013). (C) Transcript levels of *yjiS* detected via northern blot. The conditions tested are growth in LB to OD_600_ 0.2, OD_600_ 2.0, and overnight (O/N), and growth in SPI-2 medium to an OD_600_ of 0.3. 5S rRNA was probed as loading control. (D) Coverage plots (RPKM values; “RNA” = total RNA, “70S” = ribosome footprints) from Ribo-seq performed on cells grown in LB to OD_600_ = 0.4, or OD_600_ = 2.0, and in SPI-2-inducing medium to OD_600_ = 0.3. Data from Venturini et al. (2020). (E) Expression pattern of SPA-tagged YjiS detected via western blot, with GroEL probed as loading control. The growth conditions are the same as in panel C.

There are several features that should help predict a potential virulence-associated function of candidate small proteins, for example, a highly correlated expression with the genes of SPI-1 or SPI-2, and an altered ability of *Salmonella* to invade or replicate in host cells when the respective sORF is disrupted. In this regard, the 54 aa-long, uncharacterized small protein YjiS is of particular interest for several different reasons. First, according to dual RNA-seq data obtained with more than a dozen different eukaryotic cell lines, the *yjiS* gene is usually the most highly induced *Salmonella* gene after host invasion (Westermann et al. 2016). Second, disruption of the *yjiS* gene is predicted to increase bacterial loads during macrophage infection (Venturini et al. 2020), a phenotype reminiscent of that of established virulence suppressors (Hu et al. 2006, Razavi et al. 2007, Baek et al. 2009). Third, *Salmonella* YjiS contains an arginine-rich DUF1127 domain of unknown function (Fig. 1A). Recent work on DUF1127 domain-containing small proteins in α-proteobacteria reported molecular functions as RNA-binding proteins or regulators of phosphate and carbon metabolism (Grützner et al. 2023, 2021, Kraus et al. 2020, McIntosh et al. 2021). However, according to phylogenetic analysis, the YjiS proteins found in enteric bacteria such as *Salmonella* or *Escherichia coli* seem to form a different clade (Kraus et al. 2020) (Fig. 1B), raising the possibility that *Salmonella* YjiS might work by an entirely different mechanism.

In this report, we describe a first attempt to functionally characterize the YjiS small protein in the context of *Salmonella* virulence. Validating our previous global perturbation screen, we show that lack of YjiS results in higher pathogen loads during the late stages of macrophage infection. This phenotype was not the result of enhanced host cell entry or intracellular hyper-replication, but seemed to be caused by an increased re-infection of host cells upon the premature release of intracellular *Salmonella*. Dual RNA-seq-based expression profiling suggested that YjiS induces specific responses in the infected macrophages. This included the hyper-activation of oxidative stress and an upregulation of cell cortex-related gene sets in host macrophages infected with YjiS-deficient mutant bacteria. Mechanistically, we found that YjiS localizes to the inner bacterial membrane and revealed its binding partners to be primarily comprised of inner-membrane proteins, including a virulence-related two-component system that acts on flagella-mediated motility. Molecular and morphological characterization of Δ*yjiS Salmonella* mutants revealed an aberrant expression of motility genes and elevated flagella numbers. Together, this initial characterization encourages future studies into the role and mechanism of the YjiS small protein that is encoded by one of the top-induced genes in intracellular *Salmonella*.

## Methods

### Bacterial genetics and *in*-*vitro* growth conditions

Bacterial strains, plasmids, and oligonucleotides used in the present study are reported in Supplementary Table S1. Chromosomal editing (Δ*yjiS* and insertion of a YjiS-SPA tag) was achieved via recombination as previously described (Datsenko and Wanner 2000). Combination of chromosomal mutations with GFP+ was carried out via P22 phage transduction (Sternberg and Maurer 1991). All bacterial strains were routinely grown starting by a single colony inoculated in 2 ml of Lennox-Broth (LB) medium and grown overnight at 37°C with shaking at 220 rpm. The next day, the culture was diluted 1:100 in fresh medium, then grown to an OD_600_ of 2.0 [= SPI-1-inducing condition; (Kröger et al. 2013)]. At this stage, cells were collected via centrifugation for 2 min at 12 000 rpm, room temperature, and subjected to an *in*-*vitro* condition designed to mimick the environment of the *Salmonella*-containing vacuole. To this end, the cell pellet was washed twice with phosphate-buffered saline (PBS; Gibco) and once with SPI-2 medium (170 mM MES, 5 mM KCl, 7.5 mM (NH_4_)_2_SO_4_, 0.5 mM K_2_SO_4_, 1 mM KH_2_PO_4_, 8 µM MgCl_2_, 38 mM glycerol, 0.1% bacto casamino acid; pH 5.8) (Löber et al. 2006), and then diluted 1:50 in fresh, pre-warmed SPI-2 medium. Growth was then continued at 37°C with orbital shaking, either in a TECAN plate reader with absorbance (OD_600_) measurements in 10 min intervals, or in 10 ml flasks until the cultures reached an OD_600_ of 0.3. When appropriate, the medium was supplemented with antibiotics.

### Mammalian cell culture

RAW264.7 mouse macrophages (ATCC TIB-71) were cultured in RPMI (Gibco) medium supplemented with 10% fetal calf serum (FCS, Biochrom), 2 mM l-glutamine (Gibco), and 1 mM sodium pyruvate (Gibco) in T-75 flasks (Corning) as described in (Westermann et al. 2016). Cells were grown in a 5% CO_2_ humidified atmosphere at 37°C and routinely tested for mycoplasma contaminations (MycoAlert Mycoplasma Detection kit, Lonza).

### Infection assay

Infection of RAW264.7 macrophages was carried out as described previously (Venturini et al. 2020). Briefly, 2 days before infection cells were seeded in six-wells plates at a density of 2 × 10^5^ cells in 2 ml medium. The day before infection, one colony of bacteria was inoculated in 2 ml of LB medium and grown overnight at 37°C, shaking at 220 rpm. On the day of infection, macrophages were counted to calculate the number of bacteria required to reach a multiplicity of infection (MOI) of 10 (or, in case of the bacterial emergence assay and the dual RNA-seq experiment, MOI 50). Bacteria were then collected via centrifugation at 12 000 rpm for 2 min at room temperature, resuspended in 10% mouse serum in RPMI and incubated for 20 min at room temperature. After this opsonization, fresh RPMI with bacteria was used to replace the macrophage medium, the plates centrifuged for 10 min at 250 *g*, room temperature, and incubated for 20 min at 37°C in 5% CO_2_ humidified atmosphere. The medium was replaced with RPMI containing gentamicin (50 mg/ml) for 30 min, and then further replaced with medium containing a lower gentamicin concentration (10 mg/ml). At the required time points, cells were washed with PBS and incubated for 5 min at room temperature with 0.1% Triton X-100 in PBS. The lysates were then diluted in PBS and plated in technical duplicates on LB plates. These plates were incubated overnight at 37°C and colony-forming units (CFUs) of each strain counted on the following day.

### RNA extraction

Extraction of total RNA was performed using the hot phenol method. Three OD equivalents of cells were collected when grown in SPI-2 medium, while four OD equivalents of cells were collected after growth in LB medium. The cells were mixed with 0.2 vol. of stop mix solution (95% ethanol, 5% acidic phenol) and frozen in liquid nitrogen. The samples were thawed and centrifuged at 4000 rpm and 4°C for 20 min, and the pellets resuspended in 600 µl of 1× TE with 0.5 mg/ml lysozyme and 60 µl (w/v) SDS. The samples were incubated at 64°C for 2 min, after which 66 µl of 3 M NaOAc, pH 5.2 and 750 µl acidic phenol (Roth) were added and incubated for 6 min at 64°C with occasional inversion. After this, the samples were centrifuged for 15 min at 16 000 rpm and 4°C. The upper, aqueous phase was transferred to phase-lock gel tubes (Quanta bio) with 750 µl chloroform, shaken for 10 s, and centrifuged for 15 min at 16 000 rpm and 4°C. The supernatant was transferred to a new tube, mixed with three volumes of 30:1 ethanol: sodium acetate solution, and incubated for at least 1 h at −20°C. RNA was then precipitated via centrifugation for 30 min at 16 000 rpm, 4°C, washed with 70% ethanol, and resuspended in water by shaking at 700 rpm, 65°C for 5 min.

### Northern blot analysis

RNA samples were resolved via denaturing PAGE electrophoresis (6% polyacrylic acid and 7 M urea). Prior to loading, 5 µg of RNA mixed with an equal volume of 2× RNA loading buffer (0.025% [w/v] bromophenol blue, 0.025% [w/v] xylene cyanol, 18 µM EDTA pH 8, 0.13% [w/v] SDS, 95% formamide) were denatured by boiling for 5 min. The run was for 2 h at 200 V. The RNA was transferred to Hybond membranes (Fisher scientific) via wet blotting for 1 h at 50 V and 4°C, and crosslinked by exposure to 254 nm UV light at 0.12 J. The membrane was then pre-incubated with 15 ml Roti Hybri-Quick (Roth) for 30 min at 42°C, after which 2-5 pmol of gene-specific, ^32^P-labeled DNA oligonucleotide was added and hybridized at 42°C.

### Western blot analysis

Proteins samples were boiled for 5 min and resolved via 15% SDS-PAGE. The gels were then either stained with Roti-Blue Coomassie (Roth) or used for immunoblotting. For the latter, the resolved samples were transferred to a PVDF membrane (Perkin Elmer) in a semidry blotter. The membrane was blocked with 10% (w/v) milk in TBS-T for 1 h at room temperature. Thereafter, the membrane was incubated with the corresponding primary and secondary antibodies. Membranes were developed with ECL chemiluminescent solution (GE Healthcare) and visualized using the Fuji LAS-4000 imager.

### Flow cytometry

Flow cytometry analysis was performed to quantify the proportion of infected macrophages (that harbored intracellular GFP-expressing bacteria), to trace the kinetics of emerging bacteria from macrophages, and to measure the signal intensity of GFP emitted by infected host cells (as a proxy for the intracellular bacterial load). To analyze intracellular bacteria, infected cells were detached from the bottom of the wells via scraping, collected, and washed twice with cold PBS (5 min, 550 *g*, 4°C). The samples were analyzed using a MACSQuant Analyzer (Miltenyi Biotec) and FACS gates were set based on appropriate reference controls (uninfected RAW macrophages, constitutively GFP-expressing wild-type *Salmonella*, PBS only). In case of infected macrophages, 20 000 intact cells were recorded by each replicate and quantified (ratio of GFP-positive to GFP-negative cells and GFP signal intensity of GFP-positive cells, respectively). To measure extracellular bacteria that had escaped from macrophages, bacteria in a fixed volume (3 × 300 µl per time point and replicate) of the supernatants of infected cultures were pelleted and washed with PBS (2 min, 12 000 rpm, 4°C). The total numbers of free, GFP-positive bacteria per sample were enumerated on a NovoSampler Q system (Agilent). Flow cytometry data were analyzed using the FLOWJO software.

### LDH release assay

To quantify host cell death upon infection, the levels of lactate dehydrogenase (LDH) in the infection supernatant were quantified with the Cytotox 96 non-radioactive cytotoxicity assay (Promega) according to the manufacturer's instructions. The absorbance at 490 nm was measured using a Multiskan Ascent (Thermo Fisher Scientific).

### Gentamicin sensitivity assay

Gradient plates with gentamicin were prepared following the description of (Du et al. 2020). The top layer ontained 10 µg/ml and the lower layer 50 µg/ml of gentamicin.

### RNA sequencing and analysis

For dual RNA-seq, total RNA quality was checked using a 2100 Bioanalyzer with the RNA 6000 Nano kit (Agilent Technologies). The RNA integrity number (RIN) for all samples was ≥ 5.0. (Note that the RIN can be determined either in bacterial or eukaryotic mode, meaning that the respective other ribosomal RNA bands in mixed host–bacterial total RNA samples will be misclassified as degradation products by the Bioanalyzer software, explaining the relatively low RIN values even for high-quality RNA from infection samples.) DNA libraries suitable for sequencing were prepared from 500 ng of total RNA using the Illumina Stranded Total RNA Prep, Ligation with Ribo-Zero™ Plus kit (Illumina) according to the manufacturer’s instructions. After 14 cycles of PCR amplification, the size distribution of the barcoded DNA libraries was estimated to ∼330 bp by electrophoresis on Agilent DNA 1000 Bioanalyzer microfluidic chips. Sequencing of pooled libraries, spiked with 1% of a PhiX control library, was performed at a depth of 32 to 46 million reads/sample in single-end mode with 100 nt read length on a NextSeq 2000 platform (Illumina) using two P2 sequencing kits and a custom recipe with one dark cycle at the beginning of read 1. Demultiplexed FASTQ files were generated with BCL Convert v4.0.3 (Illumina).

Raw sequencing reads were quality- and adapter-trimmed via Cutadapt (Martin 2011) version 2.5 using a cutoff Phred score of 20 in NextSeq mode, and reads without any remaining bases were discarded (parameters: - -nextseq-trim=20 -m 1 -a ACTGTCTCTTATACACATCT). Processed reads were unambiguously assigned to either mouse or *Salmonella* via FastQ Screen (Wingett and Andrews 2018) version 0.15.2 with parameters - -aligner bowtie2 - -tag for read tagging and - -filter “03” and “30” to generate split FASTQ read files for mouse and *Salmonella*, respectively. The parameter - -subset 0 was applied to process all reads instead of only a subset. For this and subsequent analyses, NCBI RefSeq assemblies GRCm39 for mouse and GCF_000210855.2 for *Salmonella* were used.

For analyzing gene expression of *Salmonella*, we applied the pipeline READemption (Förstner et al. 2014) v0.4.5 to align reverse-complemented sequences of all reads longer than 11 nt to the reference genome using segemehl v0.2.0 (Hoffmann et al. 2009) with an accuracy cut-off of 95% (parameters: -l 12 -a 95 -R). We used READemption gene_quanti to quantify aligned reads overlapping genomic features by at least 10 nt (-o 10) on the sense strand (-a). For this, we applied NCBI gene annotations together with custom annotations for untranslated regions (UTRs) and small RNAs (sRNAs) (Venturini et al. 2020). Based on these counts, differential expression analysis was conducted via DESeq2 (Love et al. 2014) version 1.24.0. Read counts were normalized by DESeq2 and batch correction was applied by modelling the replicate number as a batch variable in the design formula. Fold-change shrinkage was conducted by setting the parameter betaPrior to TRUE. Differential expression was assumed at adjusted *P*-value after Benjamini–Hochberg correction (*P*_adj_) < 0.05 and |log_2_ fold-change| ≥ 1.

For the mouse data, we mapped prefiltered reads to the reference genome using STAR (Dobin et al. 2013) v2.7.2b with default parameters and exons based on RefSeq annotation version 109 for GRCm39. Read counts on exon level summarized for each gene were generated using featureCounts v1.6.4 from the Subread package (Liao et al. 2014). Multi-mapping and multi-overlapping reads were counted strand-specifically and reversely stranded with a fractional count for each alignment and overlapping feature (parameters: -s 2 -t exon -M -O - -fraction). The count output was utilized to identify differentially expressed genes using DESeq2 (Love et al. 2014) version 1.24.0. Read counts were normalized by DESeq2, and RUVs (*k* = 3) from the RUVseq package (Risso et al. 2014) version 1.32.0 was used to estimate factors of unwanted variation, which were subsequently added to the DESeq2 design formula. Fold-change shrinkage was applied by setting the parameter “betaPrior=TRUE”. The PCA plot was created from RUVs-corrected vst-transformed counts using the plotPCA function from DESeq2 based on the top 10 000 genes together with ggplot2 (Wickham 2016) version 3.4.2. Differential expression of genes was assumed at *P*_adj_ < 0.05 and |log_2_ fold-change| ≥ 0.5.

ClusterProfiler (Yu et al. 2012) version 3.12.0 was used to perform functional enrichment analyses based on Kyoto Encyclopedia of Genes and Genomes (KEGG) pathways and Gene Ontology (GO) terms. The GSEA function was applied for gene set enrichment analysis considering the DESeq2 log_2_ fold-change of all analyzed genes. Volcano plots for *Salmonella* and mouse were generated from DESeq2 results using the Bioconductor package EnhancedVolcano (Blighe 2024; doi: https://github.com/kevinblighe/EnhancedVolcano; version 1.2.0/1.16.0; accessed: 5/6/2023).

Comparative gene expression analysis of Δ*yjiS* vs. wild-type *Salmonella* grown in SPI-2-inducing medium using conventional RNA-seq was analogous to our previous assessment of the small *Salmonella* protein MgrB (Venturini et al. 2020). Briefly, cDNA libraries derived from total RNA were prepared at Vertis Biotechnologie AG after rRNA depletion [Ribo-Zero rRNA Removal Kit (Bacteria); Illumina]. Sequencing was performed on an Illumina NextSeq 500 platform with approximately 20 million reads per library. Alignment to the *Salmonella* Typhimurium SL1344 genome was performed using READemption (Förstner et al. 2014) (version 0.4.5) and differential gene expression analysis was carried out with edgeR (Robinson et al. 2010) (version 3.20.8). Only genes with at least 5 uniquely mapped reads in two experimental replicates were considered. The cut-off for differential expression was set to *q*-value < 0.05 and |log_2_ FC| > 2.

### Co-immunoprecipitation

To identify protein interactors of YjiS, a co-immunoprecipitation (co-IP) was performed on ∼60 OD equivalents of cells grown in SPI-2 medium in three biological replicates. Bacteria were collected via centrifugation (4000 rpm, 4°C for 20 min) and washed with ice-cold PBS. The pellet was resuspended in 800 µl of lysis buffer (1 mM MgCl_2_, 20 mM Tris HCl pH 7.5, 1% igepal, 1 mM DTT, 1 mM PMSF, 10 U/ml DNase I). An equal volume of 0.1 mm glass beads (Roth) was added and the samples were lysed via mechanical beating using a Mixer mill MM 400 (Retsch) for 10 min at 30 Hz. The samples were then centrifuged at 16 000 rpm and 4°C for 30 min to remove unbroken cells. The supernatant was transferred to a new 2 ml tube and centrifuged again for 10 min. The supernatant was again transferred to a new tube and an aliquot of 25 µl (input) was stored at −20°C. Twenty-five microliter of pre-washed anti-FLAG M2 magnetic beads (Sigma) were added to each sample and the tubes were incubated for 16 h, rotating at 4°C. After this step, the beads were washed three times with 500 µl of lysis buffer. The beads were then soaked in 35 µl of protein loading buffer and boiled for 5 min at 95°C to elute the pulled-down proteins. Both input and pull-down samples were separated by a Coomassie-stained 1D SDS gel. Sample preparation was performed as previously described (Bonn et al. 2014). Briefly, gel lanes were fractionated into 10 gel pieces, cut into smaller blocks, and transferred into low binding tubes. Samples were washed until gel blocks were destained. After drying of gel pieces in a vacuum centrifuge, they were covered in trypsin solution. Digestion took place at 37°C overnight before peptides were eluted in water, assisted by ultrasonication. The peptide-containing supernatant was transferred into a fresh tube, desiccated in a vacuum centrifuge, and peptides were resolubilized in 0.1% (v/v) acetic acid for mass-spectrometric analysis.

### Mass-spectrometric analysis

Tryptic peptides were subjected to liquid chromatography (LC) separation and electrospray ionization-based mass-spectrometry (MS), applying exactly the same injected volumes in order to allow for label-free relative protein quantification. To this end, peptides were loaded on a self-packed analytical column (OD 360 µm, ID 100 µm, length 20 cm) filled with Reprosil C18, 3 µm material (Dr Maisch, Ammerbuch-Entringen, Germany) and eluted by a binary nonlinear gradient of 5%–99% acetonitrile in 0.1% acetic acid over 76 min with a flow rate of 300 nl/min. LC-MS/MS analyses were performed on an LTQ Orbitrap (ThermoFisher Scientific, Waltham, Massachusetts, USA) using an EASY-nLC II liquid chromatography system. For MS analysis, a full scan in the Orbitrap with a resolution of 30 000 was followed by collision-induced dissociation of the five most abundant precursor ions. MS2 experiments were acquired in the linear ion trap.

Database search against a *Salmonella* Typhimurium SL1344 reference downloaded from Uniprot (date 23/08/2018, organism ID 216597, 4 659 entries) as well as label-free quantification were performed using MaxQuant (version 1.6.2.6) (Cox and Mann 2008). Common laboratory contaminants and reversed sequences were included in the MaxQuant software. Search parameters were set as follows: trypsin/P specific digestion with up to two missed cleavages, methionine oxidation and N-terminal acetylation as variable modification, match between runs with default parameters enabled. The false discovery rates of protein and peptide spectrum match levels were set to 0.01. iBAQ values (Schwanhäusser et al. 2011) were used to provide quantitative information on protein abundance. Only unique peptides were considered for protein quantification.

### Cell fractionation

For sub-fractionation of bacteria (adapted from (Knoke et al. 2020)), a total of ∼60 OD equivalents of cells grown in SPI-2-inducing conditions were collected via centrifugation (4000 rpm, 4°C for 20 min) and washed with lysis buffer (20 mM Tris HCl pH 7.5, 150 mM KCl, 1 mM MgCl_2_, 1 mM PMSF). After centrifugation, the supernatant was removed and the pellets frozen in liquid nitrogen for storage at −20°C. The pellets were thawed, resuspended in 4 ml of lysis buffer, and disrupted via sonication (20 s burst, 10 s rest, for 15 rounds). The samples were centrifuged at 4000 rpm at 4°C for 30 min to remove unbroken cells. The equivalent of 1 OD was collected from the supernatant (lysate). The remaining sample was diluted by addition of 4 ml and ultracentrifuged with an Optima XP-80 ultracentrifuge (SW 60 Ti rotor; Beckman Coulter) for 90 min at 150 000 *g* and 4°C. The supernatant (cytosol) was collected and stored at −20°C, while the pellet was incubated at 4°C with gentle stirring in the same volume of lysis buffer containing 0.5% N-lauroylsarcosyn to solubilize the inner membrane until the pellets were dissolved. After this, the samples were ultracentrifuged for 60 min at 150 000 *g*, 4°C. The supernatant (inner membrane fraction) was collected and stored at −20°C. The pellets were resuspended via gentle stirring at 4°C in the same volume of lysis buffer with 2% Triton X-100. After solubilization, the samples (outer membrane fractions) were stored at −20°C. 500 µl aliquots of the cytosol, the inner and outer membrane fractions were concentrated for western blot analysis. To this end, 1/10 v/v of sodium desoxycholate (0.015% w/v stock) was added and the samples incubated for 10 min at room temperature. Afterwards, 1/10 v/v of 100% trichloroacetic acid (TCA) was added and incubated for 10 min at room temperature. The samples were centrifuged for 10 min at 16 000 rpm and 4°C. The supernatant was discarded and the pellets washed three times with ice-cold 80% acetone. After the last wash, the pellets were dried and resuspended in protein loading buffer for western blot analysis.

To distinguish membrane-associated from integral transmembrane proteins, cells were processed as described above, up to the cytosol isolation step. The pellet following ultracentrifugation was then dissolved in a high-salt buffer (lysis buffer with 500 mM KCl) by stirring at 4°C. Ultracentrifugation was repeated at 150 000 *g*, 4°C for 1 h. The supernatant (membrane-associated proteins) was immediately concentrated via TCA precipitation as described above. The pellet was dissolved in lysis buffer with 1% (m/v) dodecylmaltoside to solubilize membrane-embedded proteins. The insoluble fraction was removed via ultracentrifugation (1 h at 150 000 *g* and 4°C). The supernatant was collected and concentrated as described above.

### Formaldehyde crosslinking

For crosslinking of the YjiS-SPA protein to its interactors, the equivalent of 60 ODs of cells grown in SPI-2 medium was collected by centrifugation at 4000 rpm, 4°C for 20 min and washed once with ice-cold PBS. After this, the pellet was resuspended in 1 ml of PBS and formaldehyde (Roth) was added to a final concentration of 1%, followed by an incubation for 10 min at 37°C. The crosslinking reaction was quenched by addition of glycine (Roth) to a final concentration of 0.125 M. An aliquot of the sample was collected and stored at −20°C, while the remaining sample was centrifuged at 13 000 rpm, 4°C for 2 min and resuspended in 800 µl of lysis buffer (1 mM MgCl_2_, 20 mM Tris HCl pH 7.5, 1% igepal, 1 mM DTT, 1 mM PMSF, 10 U/ml DNase I), followed by the addition of an equal volume of 0.1 mm glass beads (Roth). The samples were lysed via mechanical beating on a Mixed mill MM 400 (Retsch) for 10 min at 30 Hz. After lysis, the samples were centrifuged at 16 000 rpm for 30 min at 4°C. Half of the supernatant was removed and stored at −20°C, while the remaining half was treated with 1:5 v/v of RNase A: T1 mix for 20 min at 20°C. The digestion was stopped by adding protein loading buffer and boiling for 5 min.

### Motility assay

To assess *Salmonella* swimming capacity in a low-viscosity condition, 6 µl of a wild-type or Δ*yjiS* overnight culture were spotted at the center of 0.3% SPI-2 agar plates. The distance of swimming from the rim to the site of inoculation was measured after 6 h of incubation at 37°C.

### Electron microscopy

Overnight cultures of wild-type *Salmonella*, Δ*yjiS* bacteria, and of a Δ*fliC* mutant (as a flagella-negative control strain) were fixated in 2.5% glutardialdehyde in 50 mM cacodylate buffer containing 1 M KCl and 0.1 M MgCl_2_. Pellets of fixated samples were resuspended in 45 µl of PBS and incubated in a droplet on carbon-coated 300 mesh grids for 2 min. The liquid was removed, followed by a brief washing step in ddH_2_O. After removal of the water, grids were incubated in 0.5% uranyl acetate dissolved in ddH_2_O for 1 min. After removal of the contrasting solution, grids were dried in a gridbox. The images were taken using the JEOL JEM-1400Flash scanning transmission electron microscope (JEOL Germany, Freising) with 120 kV acceleration voltage and a Matataki camera. The ImageJ software (Schneider et al. 2012) was used to quantify the number and length of individual flagella, in form of a blinded assessment of images without prior knowledge of the corresponding genotype.

## Results

### The small protein YjiS is produced under infection-mimicking conditions

The *yjiS* gene is predicted to be coexpressed with SPI-2 genes (Hautefort et al. 2008, Kröger et al. 2013), highly transcribed during the infection of mouse macrophages (Srikumar et al. 2015) and of human epithelial cells (Westermann et al. 2016), as well as in the spleen of infected mice (Rollenhagen et al. 2004). To validate these predictions by independent experiments, we used northern blot analysis detecting the *yjiS* mRNA to confirm the upregulation of this gene in *Salmonella* grown in minimal medium mimicking the intra-vacuolar milieu (“SPI-2-inducing condition”; see “Methods” section). By contrast, the *yjiS* mRNA was not detected during growth in rich LB medium (Fig. 1C).

Interrogation of previous ribosome profiling data (Venturini et al. 2020) likewise revealed YjiS translation exclusively in the SPI-2-inducing condition, but not in rich medium (Fig. 1D). Accordingly, an epitope-tagged version of this protein that we newly generated, was readily detectable by western blot in samples from SPI-2-induced *Salmonella*, but not in samples collected from LB cultures no matter the growth stage (Fig. 1E). We conclude from these experiments that the small protein YjiS is selectively produced when *Salmonella* grows under conditions that mimic its *in*-*vivo* niche inside host cells.

### Highly conserved DIGL residues are dispensable for the virulence-suppressing function of YjiS

**Figure 2.**
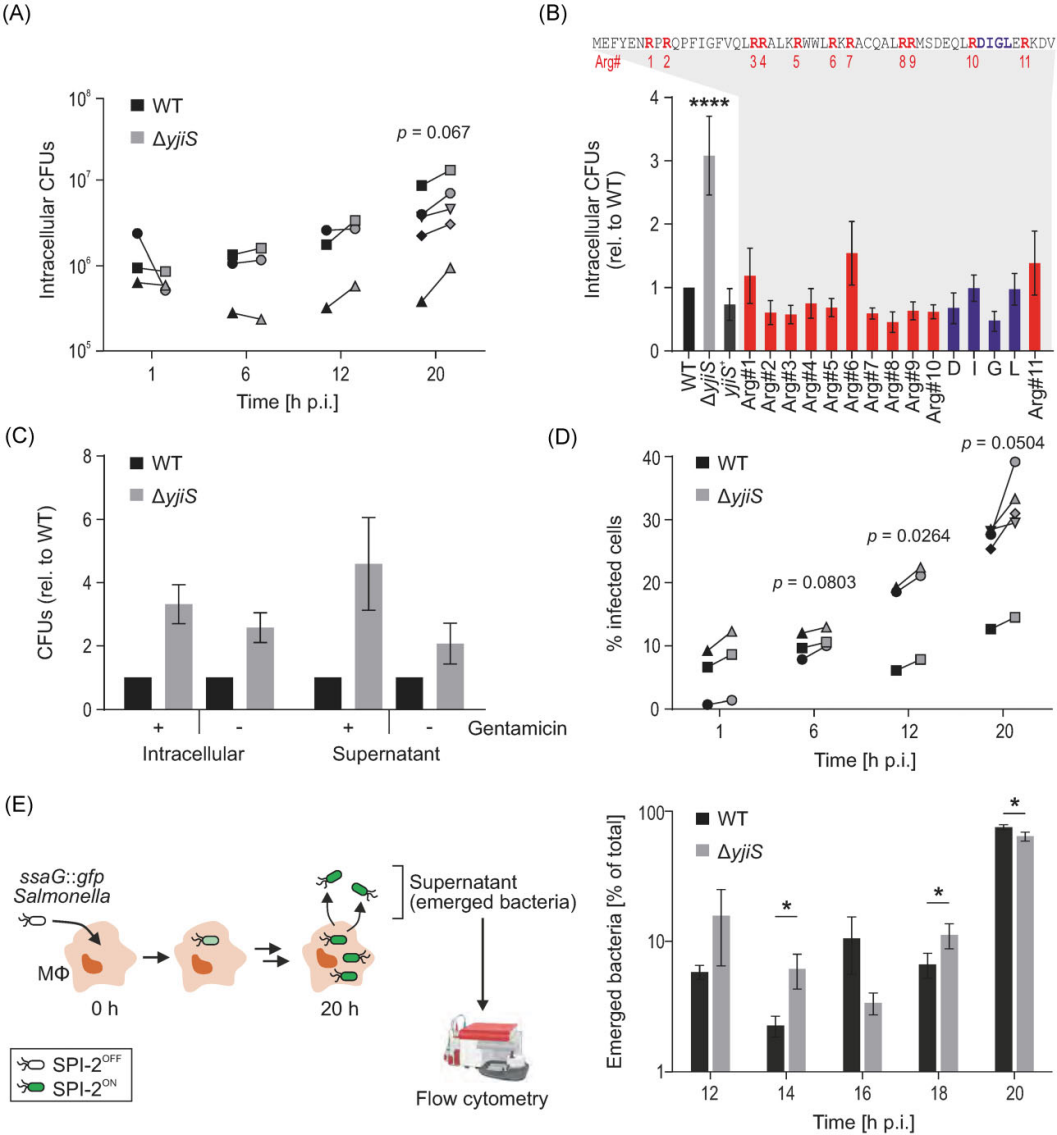
YjiS counteracts *Salmonella* virulence in a macrophage infection model. (A) Time course of RAW264.7 macrophage infection with wild-type or Δ*yjiS Salmonella* at an MOI of 10. At the indicated time points after infection, intracellular bacteria were recovered and the CFUs from five (20 h) or three (remaining time points) biological replicate experiments enumerated. *P*-values <0.1 (according to a paired *t* test) are indicated. (B) CFU assay of RAW264.7 macrophages collected at 20 h p.i. with *Salmonella* wild-type, Δ*yjiS* (carrying the empty vector control), *yjiS*^+^ (Δ*yjiS* background with the plasmid encoding wild-type *yjiS* from its native promoter), or specific *yjiS* mutants (alanine mutants of each of the 11 arginines (red) or single residues of the DIGL motif (blue)). The CFU counts from three biological replicates were normalized to those of the wild-type strain and the bars and error bars indicate the mean and standard deviation (SD), respectively. According to a one-way ANOVA analysis, the only signficant difference (*P*_adj_ value <0.0001) was between wild-type and Δ*yjiS*, while differences between the other strains and the wild-type were non-significant (*P*_adj_ value > 0.05). The amino acid sequence of YjiS is depicted above the bar chart, with the mutated arginines (R) and the “DIGL” motif labeled. (C) CFU assay performed at 20 hours after infection of RAW264.7 macrophages with either wild-type or Δ*yjiS Salmonella*. Intracellular bacteria and bacteria in the supernatant were counted separately, in the presence or absence of gentamicin in the infection medium. CFU counts were normalized to the respective wild-type sample. (D) Percentage of GFP-positive (= infected) host cells during RAW264.7 infection with constitutively GFP-expressing wild-type or Δ*yjiS Salmonella*. Infection was at an MOI of 10, data were collected from five (20 h) or three (remaining time points) biological replicates, respectively. *P*-values <0.1 (according to a paired *t* test) are indicated. (E) *Salmonella* emergence assay. Left: schematic of the assay design. Macrophages are infected with either wild-type or Δ*yjiS Salmonella* strains harboring each a transcriptional SPI-2 reporter plasmid (Westermann et al. 2016) at an MOI of 50. Continuous presence of low-concentration (10 µg/ml) gentamicin prevents bacterial overgrowth of the supernatant, while providing a temporal snapshot of bacterial emergence from infected cells. Right: kinetics of bacterial emergence from macrophages during the later stages of infection based on flow cytometry gating for GFP-positive *Salmonella* in the supernatant fraction. Data are relative to the total amounts of host-released bacteria and refer to the mean ±SD of three biological replicates. Asterisks denote time points with significant differences between Δ*yjiS* and wild-type infections according to an unpaired *t* test (*P < 0.05).

To validate the involvement of YjiS in regulating *Salmonella* virulence as predicted by our previous Transposon-Directed Insertion Sequencing (TraDIS) screen (Venturini et al. 2020), we constructed a clean deletion mutant (strain Δ*yjiS*), excising the coding sequence of *yjiS* from the *Salmonella* chromosome. We used this strain to infect murine RAW264.7 macrophages and quantified intracellular bacterial loads at several time points by plating assays. Comparing CFUs to infection with the isogenic wild-type strain, we observed increased counts with the Δ*yjiS* strain from 12 h post-infection (p.i.) onwards (Fig. 2A). To complement the missing gene, we then constructed a medium-copy plasmid expressing *yjiS* from its native promoter. Transformation of the plasmid in the Δ*yjiS* background (strain *yjiS*^+^) restored the intracellular load to that of the wild-type, which confirmed the specificity of the virulence phenotype associated with *yjiS* deletion (Fig. 2B).

This infection phenotype also provided us with a robust readout system to assess the requirement of individual arginines and the highly conserved “DIGL” (aspartic acid/isoleucine/glycine/leucine) motif (see Fig. 1A) within the DUF1127 domain of YjiS. Using the *yjiS*^+^ plasmid as a template, a series of single amino acid mutants was constructed and subjected to macrophage infection assays. Interestingly, none of those mutants phenocopied Δ*yjiS* (Fig. 2B); in other words, each of the single arginines and each of the amino acids comprising the DIGL motif was dispensable for YjiS’s virulence suppressor function in this macrophage infection system. Combined with the observation that the relative position of individual arginines varies within different DUF1127 proteins (Fig. 1A), we conclude that arginine richness *per se*, rather than specific residues might be responsible for the function of this domain.

### YjiS delays *Salmonella* escape from infected macrophages

A variety of non-mutually exclusive processes could account for the phenotype of Δ*yjiS Salmonella*. An impact on host cell adherence and entry was dismissed, as CFU counts between mutant and wild-type were similar at the early stage of infection (Fig. 2A). Faster replication of the mutant was excluded by tracing growth kinetics *in vitro* and inside macrophages, which were comparable to that of the wild-type strain (Suppl. Fig. S1a, b). Likewise, as inferred from the quantification of lactate dehydrogenase release, the rates of host cell death were indistinguishable between Δ*yjiS* and wild-type infection (Suppl. Fig. S1c).

For all our macrophage assays, a low concentration (10 µg/ml) of gentamicin was continuously present in the host cell medium to restrict *Salmonella* proliferation outside host cells. However, this concentration is insufficient to completely eradicate extracellular bacteria. In fact, we observed more extracellular Δ*yjiS* than wild-type *Salmonella* also in the supernatant of infected macrophage cultures (Fig. 2C). Determination of bacterial sensitivity to this antibiotic did not reveal an altered tolerance between wild-type and Δ*yjiS* strains (Fig. S1d). Furthermore, an increased bacterial load of Δ*yjiS* was observed even when CFU assays were performed in the absence of gentamicin (Fig. 2C); there was also a slight increase in the proportion of infected cells compared to wild-type infection (Fig. 2D).

Lastly, to directly test the possibility of YjiS delaying the host release of intracellular *Salmonella*, we devised an infection assay that allowed us to trace the kinetics of pathogen emergence from infected macrophages. We focused on the later stages of the infection (12–20 h p.i.), when we started to detect an increase of Δ*yjiS* CFUs (Fig. 2A) and Δ*yjiS*-infected macrophages (Fig. 2D) as compared to wild-type infections. We based the emergence assay on an established SPI-2 reporter (plasmid p*ssaG:: gfp*; Westermann et al. 2016); as SPI-2 activity is switched on only upon host cell entry, a fluorescent signal emitted by extracellular *Salmonella* in the supernatant allows for the selective quantification of host-escaped bacteria by flow cytometry (Fig. 2E, left). In the case of *Salmonella* mutants devoid of YjiS, we indeed observed an early onset of macrophage escape as compared to wild-type kinetics (Fig. 2E, right). Altogether, these observations suggest that YjiS plays a role in restricting *Salmonella* evasion from host macrophages, thus likely reducing the re-infection of bystander cells.

### Host processes modulated by *Salmonella* YjiS in infected macrophages relate to oxidative stress and the cell cortex

**Figure 3.**
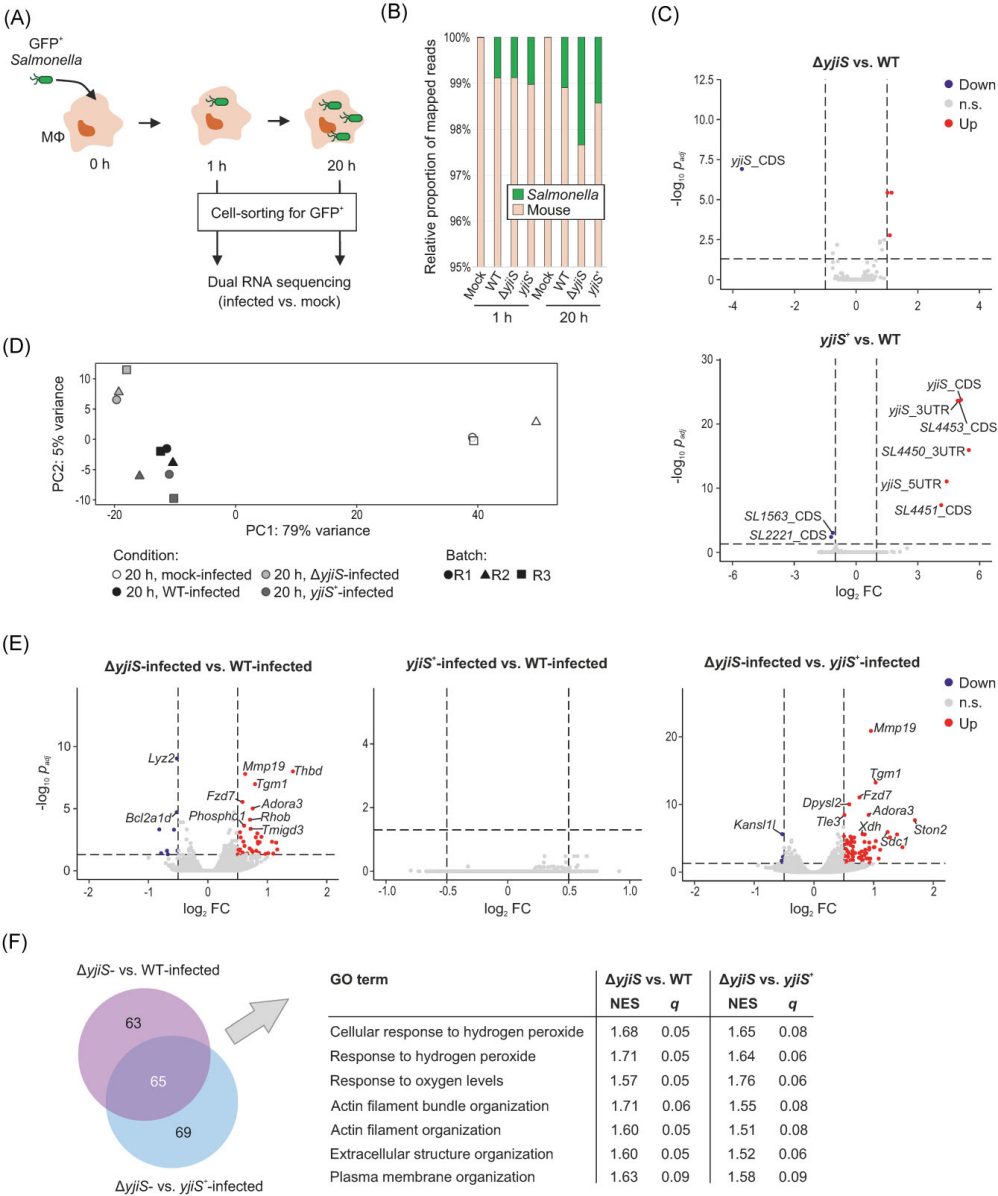
Dual RNA-seq uncovers the effect of YjiS on the macrophage response to *Salmonella* infection. (A) Experimental outline. Constitutively GFP-expressing *Salmonella* were used to infect RAW264.7 macrophages (MOI 50). At 1 and 20 h p.i., GFP-positive macrophages were enriched via cell-sorting and subjected to dual RNA-seq. (B) Mapping statistics of a representative replicate (out of 3). (C) Differential expression analysis of *Salmonella* genes (volcano plot, 20 h p.i.) for the indicated comparisons. Genes *SL4450* and *SL4451* are encoded upstream, and *SL4453* downstream of *yjiS*. “n.s.”, not significant (i.e. *P*_adj_ > 0.05 or |log2 fold-change| < 1). (D) PCA plot for the mouse data subset at 20 h p.i. (after RUVs, *k* = 3). (E) Differentially expressed mouse genes (volcano plot, 20 h p.i.). “n.s.,” not significant (i.e. *P*_adj_>0.05 or |log2 fold-change| < 0.5). (F) Functional analysis of the host response. Venn diagram depicts the overlap of Gene Ontology (GO) terms enriched between the comparisons Δ*yjiS* vs. WT infection and Δ*yjiS* vs. *yjiS*^+^ infection (set size > 50; normalized enrichment score [NES] > 1.5; *q* value < 0.1). Pathways contained in the overlap and mentioned in the main text are given in the table to the right.

In an effort to identify host processes affected by YjiS activity, we performed a comparative dual RNA-seq experiment (Westermann et al. 2016). To this end, RAW264.7 macrophages were infected with wild-type *Salmonella*, the Δ*yjiS* mutant, or the corresponding *trans*-complemented bacteria. Total RNA was extracted at 1 h or 20 h p.i. and subjected to cDNA library preparation and Illumina sequencing (Fig. 3A). The relative RNA-seq read counts that mapped to the murine and bacterial genome (Fig. 3B; Suppl. Fig. S2a) reflected the increased pathogen load in YjiS-deficient infections as observed in the CFU measurements above (Fig. 2A, B).

Calculating gene expression changes on the bacterial side, the only significantly differentially expressed genes between the three strains at matched time points were *yjiS* itself and—due to polar effects in the *yjiS*^+^ strain—its flanking genes (Fig. 3C, Suppl. Fig. S2b). On the host side, the immediate response (1 h p.i.) to the infectious stimulus was subtle and YjiS-independent (Suppl. Fig. S2c) in line with *yjiS*’s very low expression at the early time point (Suppl. Fig. S2b, upper). However, at 20 h p.i., infected macrophages segregated from mock-treated controls in a principal component analysis (Fig. 3D). What is more, Δ*yjiS*-infected samples clustered away from wild-type and *yjiS*^+^ samples (Fig. 3D), yielding a YjiS-dependent host response signature. Indeed, while not a single host gene was differentially expressed between cells infected by wild-type and YjiS-complemented *Salmonella* at 20 h p.i., differences arose when comparing either condition to Δ*yjiS*-infected macrophages (Fig. 3E).

Functional analysis revealed pathways related to oxygen stress (GO terms “Response to hydrogen peroxide” and “Response to oxygen levels”; KEGG ID “HIF-1 signaling pathway”) to be shared between the comparisons “Δ*yjiS*/WT” and “Δ*yjiS*/*yjiS*^+^” and amongst the most overrepresented YjiS-dependent host processes (Fig. 3F). Besides, several host genes (*Sdc1, Ston2, Thbd, Thbs3*) and pathways (GO terms “Actin filament organization,” “Extracellular structure organization,” and “Plasma membrane organization”) implicated in cytoskeletal and cell surface modulation were selectively upregulated in the absence of YjiS (Fig. 3E, F). Taken together, the RNA-seq data suggest that YjiS has only subtle effects on the transcriptome of intra-macrophage *Salmonella*, yet its strong upregulation induces specific host responses. These responses—when interpreted in the context of the above infection assays (Fig. 2)—seem to culminate in the suppression or delay of pathogen escape from infected macrophages.

### YjiS associates with the cytoplasmic membrane

**Figure 4.**
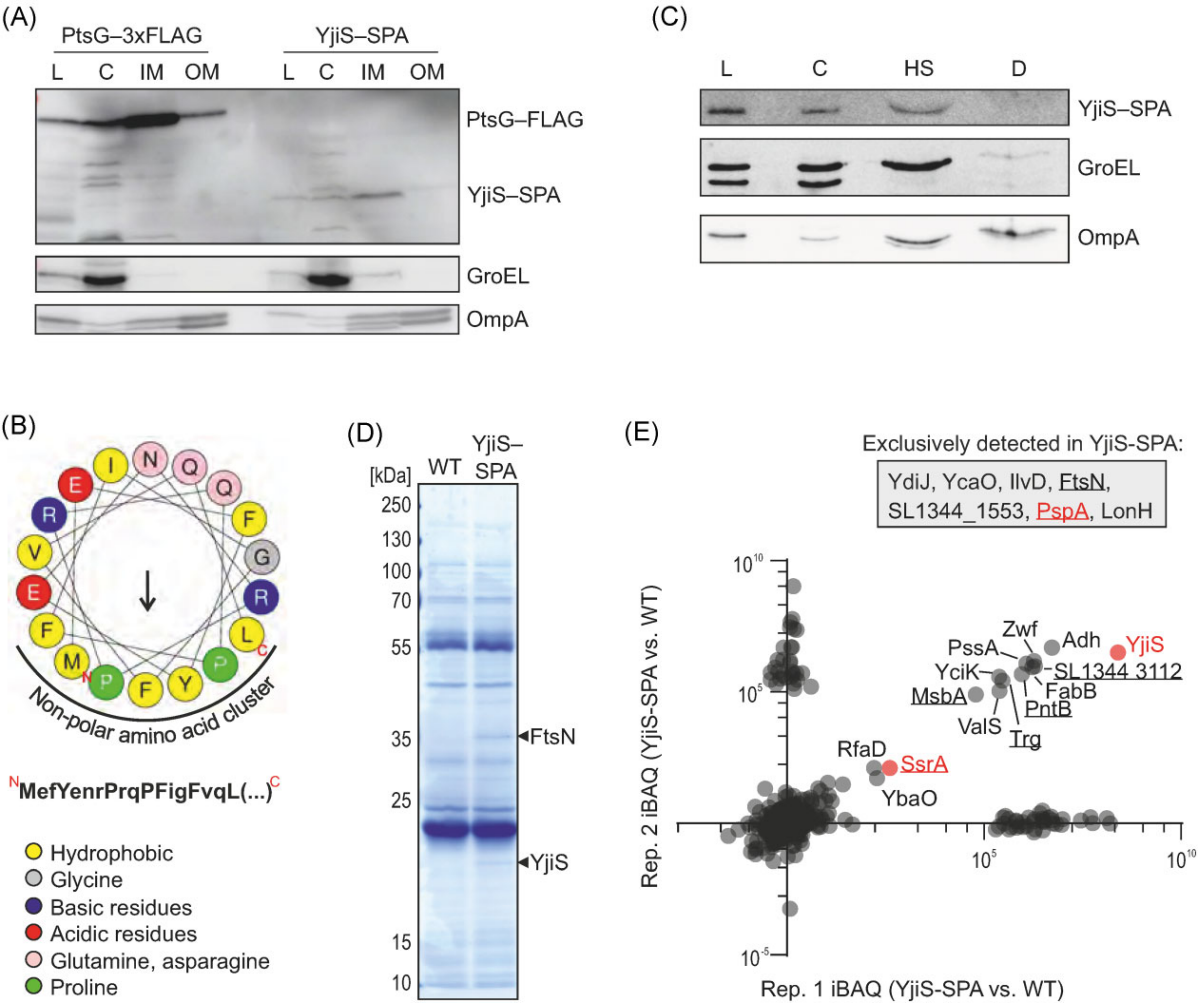
YjiS associates with inner membrane proteins. (A) Cell fractionation of *Salmonella* encoding either PtsG-3xFLAG (included as inner membrane control (Papenfort et al. 2012)) or YjiS-SPA. Bacterial fractions were separated into cytosol (C), inner membrane (IM), and outer membrane (OM), with the lysate (L) loaded as control. The western blots were probed with antibodies against FLAG (which also detect the SPA tag), against GroEL (cytosolic control), and OmpA (outer membrane control). (B) Helical wheel projection of the first 18 amino acids of the *Salmonella* YjiS protein. Hydrophobic residues are yellow, basic and acidic residues are blue or red, respectively, asparagine and glutamine are pink, prolines are green, and glycine is gray. The methionine residue marked with “N” represents the N-terminal end of the putative amphipathic helix. The arrow indicates the magnitude and direction of the hydrophobic moment. The prediction was performed using the HELIQUEST web server (Gautier et al. 2008). The amino acid consensus sequence is depicted below the helix representation, with non-polar residues capitalized. (C) Refined cell fractionation for the YjiS-SPA-tagged strain. In this case, the total membrane fraction was first treated with high-salt buffer (HS) and then with a detergent (D) to distinguish between proteins loosely interacting with the membrane and proteins inserted into the membrane. GroEL and OmpA were again probed as cytosolic and outer membrane controls. (D) Coomassie-stained SDS-PAGE of a representative co-IP performed on SPA-tagged YjiS-encoding *Salmonella* or the wild-type (untagged) control. The enriched proteins visible by naked eye and marked with a black arrowhead are YjiS itself and, putatively, FstN (as inferred from its molecular weight (36 kDa) and from the mass spectrometry-based enrichment (see panel D)). (E) Plot showing the enrichment (iBAQ difference) of proteins between the YjiS-SPA-tagged strain and the corresponding wild-type control, in each of the two replicates. The seven proteins listed in the rectangle on the top right were exclusively detected in the SPA-tagged strain, but not in the wild-type control (hence, infinitely enriched). Proteins known to localize to the membrane are underlined. The PspA and SsrA proteins, which were functionally followed up (Fig. 5; Suppl. Fig. S4), and YjiS itself are labelled in red. Dots clustering near the *x*- or *y*-axis derive from proteins that were abundantly detected in only one of the replicates, probably because of their unspecific pulldown.

We next sought to dissect the molecular basis of the YjiS-related infection phenotype. Cellular sub-fractionation using the SPA-tagged YjiS strain grown in minimal SPI-2-inducing medium coupled to western blot analysis detected the small protein in the inner membrane fraction (Fig. 4A). *In*-*silico* inspection of YjiS’s amino acid sequence using TMHMM (Krogh et al. 2001) did not propose a transmembrane domain; however, HELIQUEST (Gautier et al. 2008)—a web server to screen for amino acid sequences with specific alpha-helical properties—predicted a contiguous cluster of seven non-polar amino acids in the N-terminal alpha helix of YjiS (Fig. 4B).

To experimentally assess how YjiS associates with the membrane, we treated the membrane fraction in two consecutive steps: first, with a high-salt buffer to detach proteins loosely associated with, but not integrally inserted into, the membrane. Second, with a detergent to solubilize and recover integral transmembrane proteins. YjiS disassociated from the membrane fraction already during the high-salt treatment (Fig. 4C). We conclude that the inner membrane localization of YjiS may be due to the amphipathic alpha helix at its N-terminus, which would facilitate association of this small protein with integral transmembrane protein(s) at the cytosolic side of the inner membrane (Drin and Antonny 2010).

### Candidate interaction partners of YjiS are enriched for inner membrane proteins

Grad-seq data reporting in-gradient distributions of a bacterium’s molecular complexes (Smirnov et al. 2016) lend themselves as a resource to predict the involvement of small bacterial proteins in macromolecular assemblies (Venturini et al. 2020). Unfortunately, the Grad-seq data currently available for *Salmonella* stem from conditions in which YjiS is not expressed. As an alternative to uncover molecular interactor(s) of YjiS, we utilized expression of the SPA-tagged YjiS-variant (Fig. 1E) for co-immunoprecipitation (co-IP) (Fig. 4D) and subjected the eluted fraction to mass spectrometry. Seven proteins, namely FtsN, LonH, PspA, YdiJ, YcaO, IlvD, and SL1344_1553, were exclusively detected in the eluate of the tagged strain, but not in the control sample from analogously treated untagged *Salmonella* (Fig. 4E). Several additional proteins were selectively enriched in the YjiS co-IPs (Fig. 4E), with many of them (underlined in Fig. 4E) predicted to localize to the inner membrane.

It is possible that the above-predicted interactions of YjiS are indirect. For example, the DUF1127-containing small protein of *Rhodobacter sphaeroides* binds RNA (Grützner et al. 2021), raising the possibility that the predicted YjiS interaction with proteins was bridged by RNA. Therefore, we tested for potential interactions of YjiS with cellular RNAs using formaldehyde crosslinking in cell lysates of *Salmonella* expressing SPA-tagged YjiS. Although the crosslinking led to the expected shift of YjiS to higher molecular weight fractions (as resolved by SDS-PAGE), treatment of these samples with RNase A did not dissociate these putative YjiS-containing complexes, suggesting that YjiS-protein interactions occur in the absence of RNA (Suppl. Fig. S3). Together, this implies that *Salmonella* YjiS does not act as an RNA binder and further suggests that the function of this small protein may fundamentally differ from those of α-proteobacterial DUF1127-containing proteins.

### Towards a mechanistic understanding of YjiS in *Salmonella* virulence

Given the YjiS phenotype above, we sieved the interactome data for proteins with a known role in *Salmonella* virulence. The bacteriophage shock protein PspA, for example, is required to import metal ions needed to sustain *Salmonella* growth in the nutrient-limited environment inside host cells (Karlinsey et al. 2010, Darwin 2013). As PspA was enriched in the YjiS co-IP, we combined the Δ*yjiS* mutant with the deletion of the *psp* operon (strain Δ*yjiS*Δ*psp*) and transformed this strain with either a *yjiS* expression (*yjiS*^+^) or control plasmid. The resulting strains were used to infect macrophages and CFUs were enumerated at 20 h p.i. However, as shown in Suppl. Fig. S4a, the virulence phenotype associated with Δ*yjiS* was independent of PspA.

**Figure 5.**
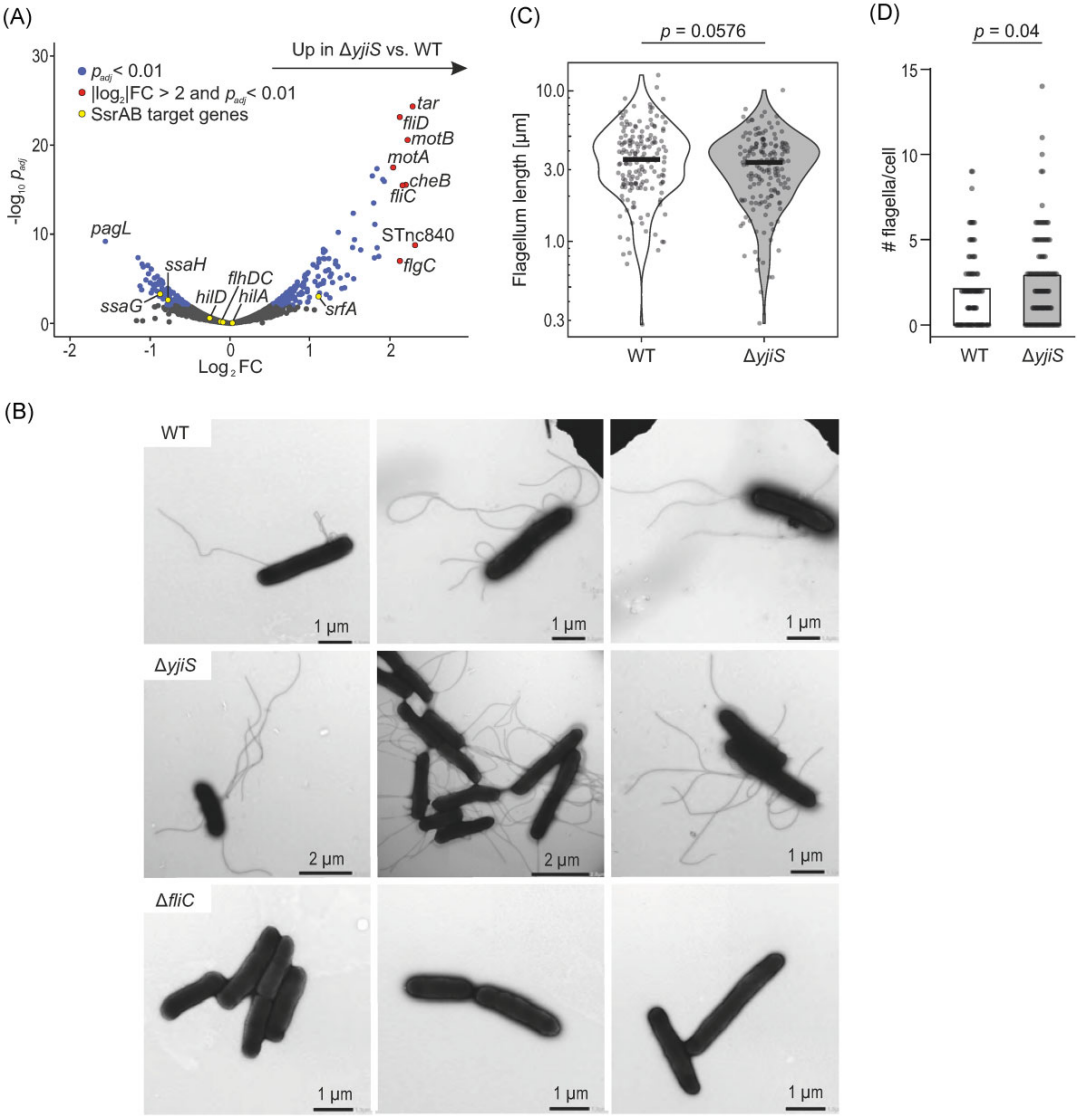
YjiS acts as a suppressor of *Salmonella* flagellum production. (A) Differential expression of coding and noncoding genes in Δ*yjiS* compared with wild-type *Salmonella* grown in SPI-2 medium to an OD_600_ of 0.3. RNA-seq data were generated from two biological replicates and are colored based on log_2_ fold-change and *P*-value adjusted for the false discovery rate. The yellow dots denote selected targets of the SsrA/B TCS, shown exemplarily for major SsrA/B-governed virulence programs, namely SPI-1 (*hilA, hilD*), SPI-2 (*ssaG, ssaH*), and flagellar genes (*flhC, flhD, srfA*) (Worley et al. 2000, Tomljenovic-Berube et al. 2010, Pérez-Morales et al. 2017). STnc840 is a verified sRNA derived from the 3’UTR of the *flgL* gene (Chao et al. 2012) and *pagL* encodes a PhoP/Q-induced lipase (Trent et al. 2001). The most regulated genes (red) are related to chemotaxis (*tar, cheB*) and motility (*fliCD, motAB, flgC*). For better data visualization, the *x*-axis was limited to a |log_2_FC| ≥ 2 and therefore excludes *yjiS*, which has a log_2_FC of -11.9 (*P _adj_* = 2.25e^−132^). (B) Visualization of bacterial flagella by electron microscopy. For each indicated strain, three representative images are depicted. *Salmonella* mutants deficient for flagellin (i.e. the major flagellum subunit; Δ*fliC*) were included as a non-motile control strain. (C, D) Quantification of the average flagellum length (C) and number of individual flagella per bacterium (D) of wild-type and Δ*yjiS Salmonella* based on electron microscopy data as shown in panel B. Quantification was performed in a blinded manner and is based on 85 or 97 individual wild-type or mutant bacteria, respectively. A Mann–Whitney test was used to assess the quantifications in panels C and D, and the *P*-values are given.

Another protein enriched in the YjiS co-IP and involved in virulence is SsrA, the sensor kinase of the SsrA-SsrB two-component system (TCS) and chief inducer of SPI-2 genes (Cirillo et al. 1998, Pérez-Morales et al. 2017). To assess whether the interaction between YjiS and SsrA would impact on the expression of SsrA/B target genes, we compared the transcriptomes of wild-type and Δ*yjiS Salmonella* grown *in vitro*, under the SPI-2-inducing condition, via RNA-seq (Fig. 5A). Overall, we observed subtle expression changes, which might explain why they had been missed by the dual RNA-seq experiment. *In vitro*, in the absence of YjiS, an operon encoding structural components of the SPI-2-associated type-III secretion system (*ssaGH*), whose transcription strictly depends on SsrB (Löber et al. 2006, Yoon et al. 2009, Tomljenovic-Berube et al. 2010), was mildly downregulated. In contrast, the SsrB-repressed genes *hilA* and *hilD* (both associated with SPI-1; Ellermeier and Slauch 2007) and *srfA* (associated with flagellar genes; Frye et al. 2006) were either not affected or de-repressed, respectively, in the absence of YjiS in this experimental condition. The most affected genes, however, are involved in motility and were up-regulated in Δ*yjiS* compared to wild-type bacteria.

We decided to follow up on the aberrant motility gene expression in the Δ*yjiS* mutant. In a first, crude assay, we did not observe major differences in the swimming ability between wild-type and Δ*yjiS Salmonella* on agar plates (Suppl. Fig. S4b). To resolve more subtle differences related to bacterial flagella, we visualized *Salmonella* cell surface structures at high resolution by electron microscopy (Fig. 5B). Quantitative image analysis revealed that bacteria produced significantly more flagella in the absence of YjiS, whereas the average flagellum length was hardly influenced by this small protein (Fig. 5C, D). Taken altogether, our initial characterization of *Salmonella* YjiS therefore suggests that this inner membrane-associated small protein carries out a function as a suppressor of flagella production and, consequently, pathogen escape from infected host cells.

## Discussion

Bacterial small proteins have long been neglected in molecular microbiology, but are now gaining center stage (Storz et al. 2014, Duval and Cossart 2017, Makarewich and Olson 2017, Orr et al. 2020, Gray et al. 2022). Previously, we performed a global approach, mining various orthogonal high-throughput datasets to predict virulence-associated small proteins in *Salmonella* Typhimurium (Venturini et al. 2020). This led to the discovery of a new role of the small protein MgrB in *Salmonella* motility and infection of epithelial cells and macrophages (Venturini et al. 2020). Additionally, our screen suggested the 54-aa YjiS as a novel virulence-associated small protein in this pathogen. That is, its mRNA was exceptionally highly up-regulated during the infection of various cell types, while transposon-mediated disruption of the *yjiS* ORF correlated with an elevated bacterial burden in infected macrophages (Venturini et al. 2020). Collectively, these predictions rendered YjiS a prime candidate for functional characterization, which we initiated with the present study.

### YjiS may be part of *Salmonella*’s stealth strategy during infection

**Figure 6.**
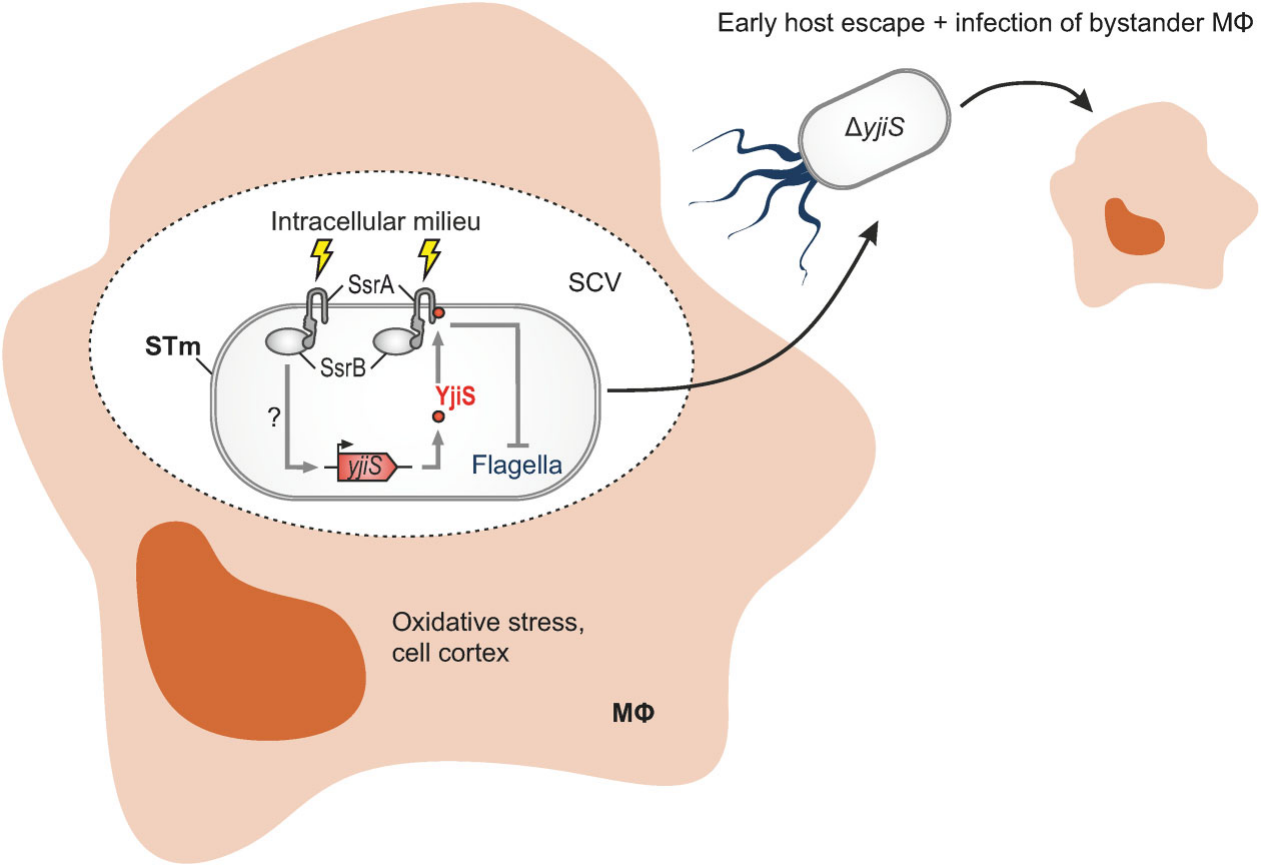
Working model of the proposed role of YjiS in *Salmonella* virulence. Upon infection of macrophages (MΦ), *Salmonella* Typhimurium (STm) resides within a *Salmonella*-containing vacuole (SCV) and activates *yjiS* transcription, potentially through the SsrA/B TCS. The YjiS protein associates with the cytoplasmic membrane, where it interacts with PspA (not depicted) and SsrA. Depletion of YjiS enhances the expression of flagellar genes and results in an increased number of individual flagella per bacterial cell. Macrophages infected with Δ*yjiS Salmonella* respond with an enhanced oxidative stress and cell cortex response, which is accompanied by an early onset of host cell escape and emergence of bacteria in the extracellular space, where they are primed to infect bystander cells.

Here, newly generated clean *yjiS* deletion and corresponding *trans*-complementation strains of *Salmonella* allowed us to confirm our transposon-based prediction and showed that, in a mouse macrophage infection model, both intra- and extracellular bacterial loads were reduced in a YjiS-dependent manner. Closer inspection of this phenotype excluded the possibilities that YjiS represses intra-macrophage replication, affects host cell death or bacterial tolerance to gentamicin. Instead, YjiS seems to help counteract the dissemination of *Salmonella* by delaying its escape from the intracellular niche (Steele et al. 2016, Flieger et al. 2018). While dampening spreading and re-infection at the cellular level in our simplistic macrophage infection system, *in vivo* such activity might be part of *Salmonella*’s stealth strategy, avoiding overt induction of host immunity and benefitting the pathogen at the population level (Fig. 6). For example, since the flagellar component FliC represents a major antigen recognized by the innate immune system (Hurley et al. 2014), there may be selective pressure on pathogens to minimize its expression during infection. Our comparative dual RNA-seq results point in a similar direction, as macrophages infected with *Salmonella* devoid of YjiS showed signs of an increased oxidative burst and overexpression of genes involved in cell cortex- and plasma membrane-related processes relative to cells infected with YjiS-proficient bacteria. Upon egress from their protective intramacrophage niche, in an *in*-*vivo* context, these extracellular *Salmonella* might become visible to neutrophils and effectively killed (Conlan 1997, Burton et al. 2014). This is in line with data from another transposon-based screen of *Salmonella* infection in different animal models, which revealed lack of *yjiS* to be correlated with impaired infection of chickens, pigs, and cattle (Chaudhuri et al. 2013). However, validating the hypothesis of a YjiS-mediated stealth mechanism of *Salmonella* requires further investigation.

### YjiS interacts with—and potentially influences the activity of—the inner membrane TCS SsrA/B

A recurrent theme with bacterial small proteins is their frequently observed localization to the cell membrane (Yadavalli and Yuan 2022). In pathogenic bacteria, several small membrane proteins have been found to be involved in the control of virulence programs (Garai and Blanc-Potard 2020). This often occurs via physical interaction with, and modulation of the activity of, membrane-standing signal receptors such as TCS. The major virulence-activating TCS in *Salmonella* spp. is PhoP–PhoQ (Groisman 2001) and there are several small proteins known to modulate PhoP/Q activity. For example, the small protein MgrB directly inhibits the autokinase activity of the PhoQ sensor histidine kinase (Lippa and Goulian 2009, Yadavalli et al. 2020), while the 34 aa-protein UgtS interacts with both PhoQ and UgtL, the latter of which is an enhancer of PhoQ autophosphorylation (Salvail et al. 2022). In both cases, this results in a reduced ratio of the phosphorylated (active) to non-phosphorylated (inactive) form of the response regulator PhoP. Since transcription of both small proteins is itself driven by PhoP/Q, their activities constitute effective negative feedback control of this central virulence program.

In the present study, cellular sub-fractionation revealed YjiS to localize to the inner membrane. As implied by computational prediction of physicochemical properties, this might be facilitated by an amphipathic helix at the protein’s N-terminus. A similar structure has recently been proposed to be responsible for the membrane association of the cyanobacterial small protein AtpΘ (Song et al. 2022). In line with its localization at the cytoplasmic membrane, interactome profiling suggested YjiS to associate with integral transmembrane proteins. Two of its potential binding partners, namely the sensor kinase SsrA and the phage-shock protein PspA, were further investigated. On the one hand, YjiS’s interaction with PspA could not be linked to the observed macrophage infection phenotype of the *Salmonella* Δ*yjiS* mutant. That is, the expression of *yjiS* from a plasmid in a Δ*psp*Δ*yjiS* background recapitulated the behavior of wild-type *Salmonella* during macrophage infection. Obviously, this does not exclude the possibility of the YjiS-PspA interaction contributing to other aspects of *Salmonella* physiology or pathogenicity.

On the other hand, we observed certain effects on SsrA/B regulon members in the absence of YjiS. While SsrA/B acts as the master activator of SPI-2 genes, it represses SPI-1, biofilm, and motility regulons (Desai et al. 2016, Pérez-Morales et al. 2017, Ilyas et al. 2018). Specific SsrB-activated SPI-2 genes (*ssaGH*) were mildly down-regulated in Δ*yjiS Salmonella*, while the expression of the SsrB-repressed *srfA* gene went up. Besides, we observed a global induction of motility genes and an increased flagella production in the absence of YjiS. However, it remains unclear to what extent these latter effects might be accounted to SsrA/B, which is known to dampen flagella gene expression, yet through the transcriptional repression of the flagellar master regulator FlhDC (Ilyas et al. 2018) whose mRNA levels were not affected by *yjiS* deletion in our RNA-seq experiment (Fig. 5A). Future investigations should therefore address whether YjiS exerts its influence on *Salmonella* pathogenicity primarily through SsrA/B and, if so, how the small protein may alter the activity of this TCS. Of note, there is a putative SsrB DNA recognition motif (Tomljenovic-Berube et al. 2010) ∼50 bp upstream of the transcription start site of *yjiS* and *yjiS* expression was blunted in a Δ*ssrB Salmonella* mutant strain (Colgan et al. 2016) (Suppl. Fig. S5). This suggests that transcriptional activation of YjiS might be governed by its target TCS, which could represent another example of small protein-mediated transcriptional feedback control (Fig. 6). Either way, the existence of YjiS homologs in Enterobacterales that lack SsrA/B (e.g. *E. coli* (Fig. 1B)), hints at additional functions of this small protein and suggests YjiS may have been secondarily coopted into the pathogenic lifestyle of *Salmonella*.

### Bacterial DUF1127 proteins show divergent functions

The DUF1127 domain is found in several confirmed and predicted bacterial small proteins. In some cases, it makes up their C-terminus (as in *Salmonella* YjiS), whereas other small protein candidates completely consist of just this domain (Mistry et al. 2021). Three small DUF1127-containing proteins from *Agrobacterium tumefaciens* play a crucial role in the phosphate metabolism of this plant pathogen (Kraus et al. 2020). Besides, recent studies implicated the *Rhodobacter* DUF1127-containing small protein CcaF1 in RNA-related processes that promote formation of photosynthetic complexes (Grützner et al. 2023, 2021). The RNA binding capacity of CcaF1 was associated with the arginine residues within DUF1127. In the present study, pulldown experiments with *Salmonella* YjiS did not provide evidence for association of this protein with cellular transcripts. In addition, mutagenesis experiments indicated that neither the arginine residues of YjiS—at least when mutated individually—nor the conserved “DIGL” motif contributed to the virulence phenotype of Δ*yjiS* in macrophages. Thus, the functions of bacterial DUF1127 domains seem to be influenced by the respective protein and organismic context.

In summary, the present work identified the small protein YjiS as a new, negative regulator of *Salmonella* virulence in a macrophage cell culture model. Together with the YjiS-dependent host responses and the YjiS interactome data, this should form a solid base for future studies towards a mechanistic understanding of this small protein in *Salmonella* infections. As encouraged by our study, a detailed understanding of YjiS could lead to direct applications, e.g. of this small protein as a drug target or antigen for vaccine development (Rollenhagen et al. 2004).

## Supplementary Material

uqae026_Supplemental_Files

## Data Availability

RNA-seq data were deposited to the Gene Expression Omnibus and are accessible under the identifier GEO GSE255513. Proteomics data were deposited to the ProteomeXchange Consortium via the PRIDE partner repository (Vizcaíno et al. 2016) and are accessible under the identifier PXD048449.
