# Supplementary figures and images for: Functional characterization of the DUF1127-containing small protein YjiS of Salmonella Typhimurium
Source: Microlife. 2025 Jan 3;6:uqae026. doi: 10.1093/femsml/uqae026 (PMC11707872; doi:10.1093/femsml/uqae026)

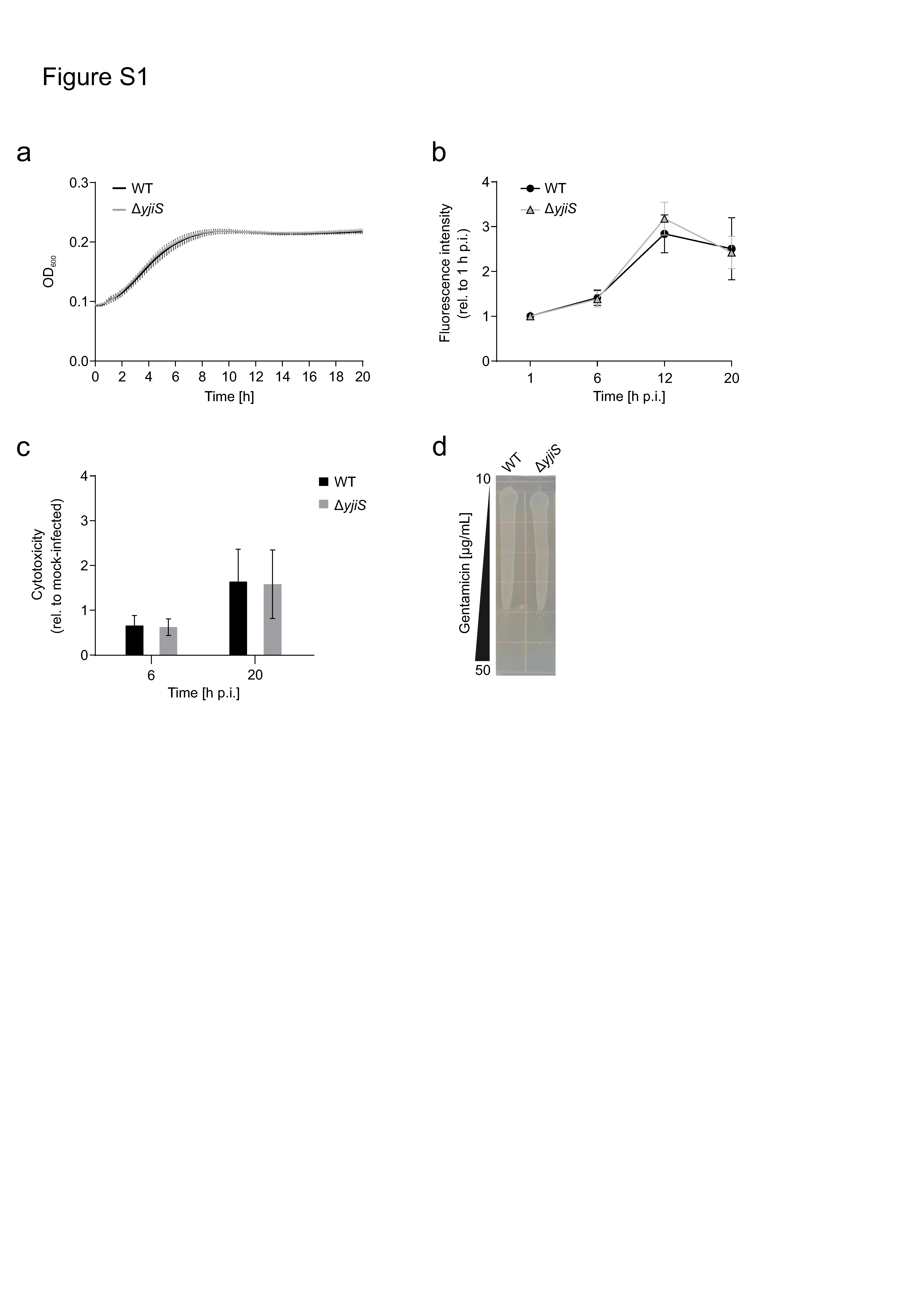

Supplement: uqae026_Supplemental_Files [file uqae026_supplemental_files.zip › Supplementary Figure_1.tif]

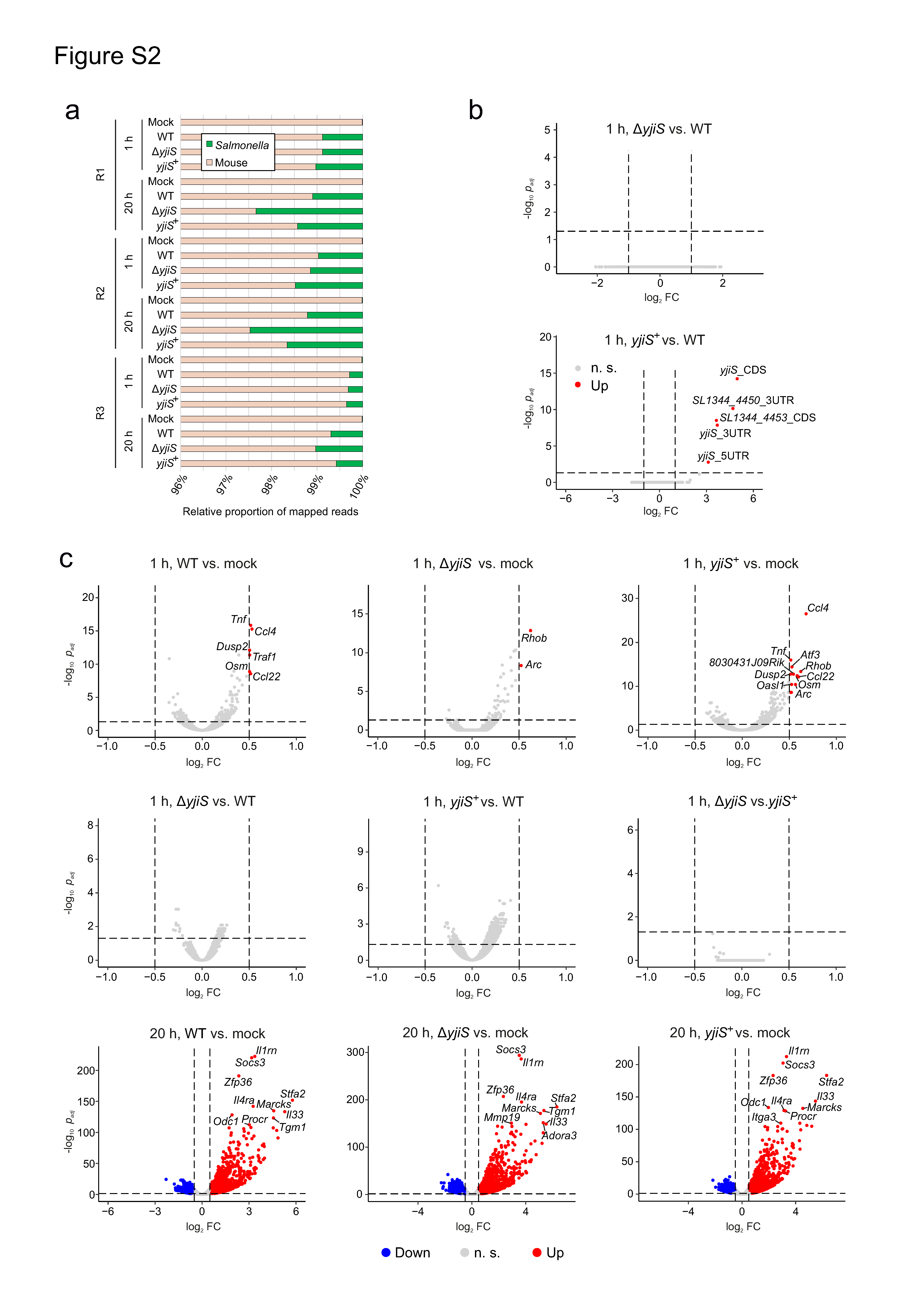

Supplement: uqae026_Supplemental_Files [file uqae026_supplemental_files.zip › Supplementary Figure_2.tif]

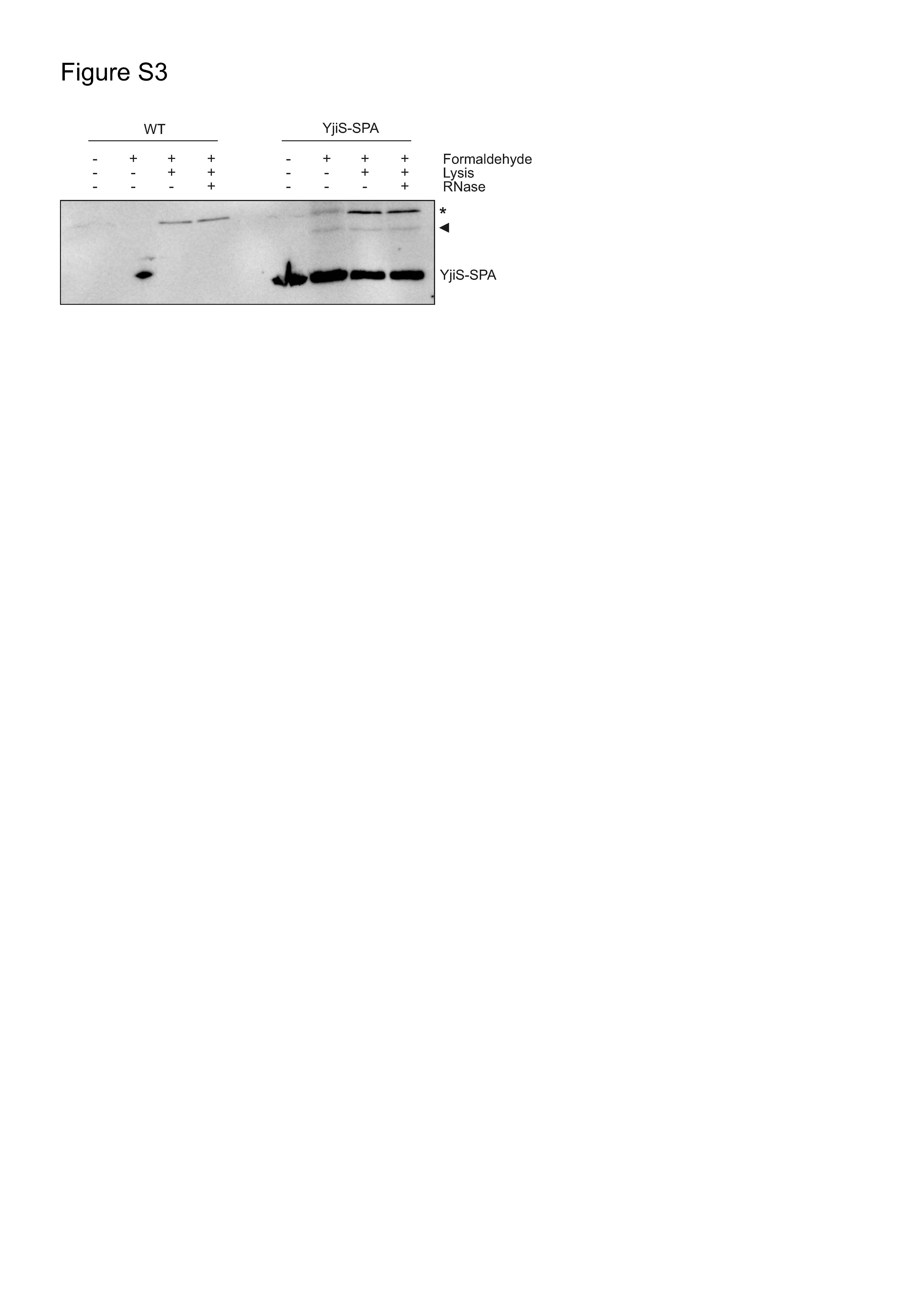

Supplement: uqae026_Supplemental_Files [file uqae026_supplemental_files.zip › Supplementary Figure_3.tif]

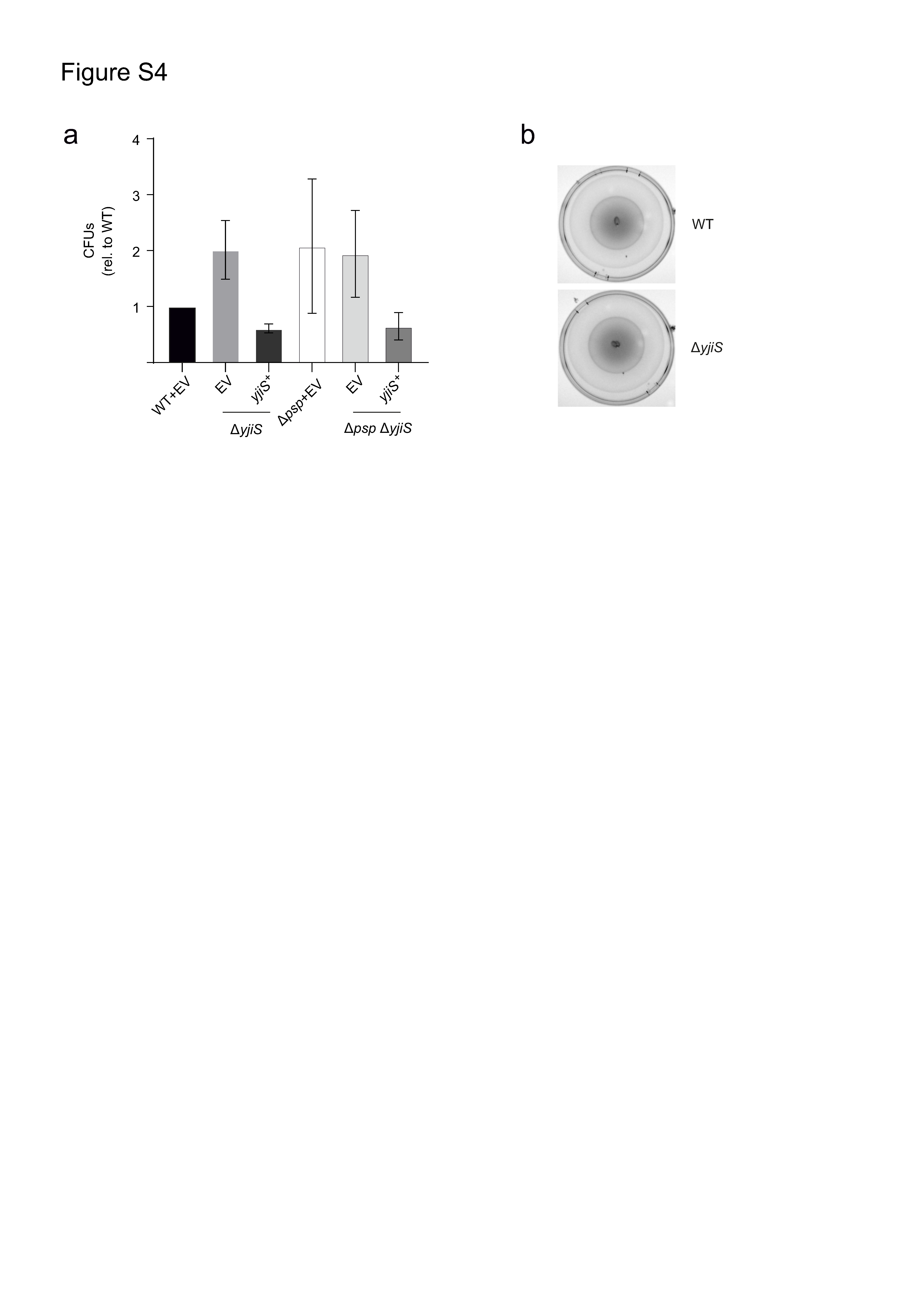

Supplement: uqae026_Supplemental_Files [file uqae026_supplemental_files.zip › Supplementary Figure_4.tif]

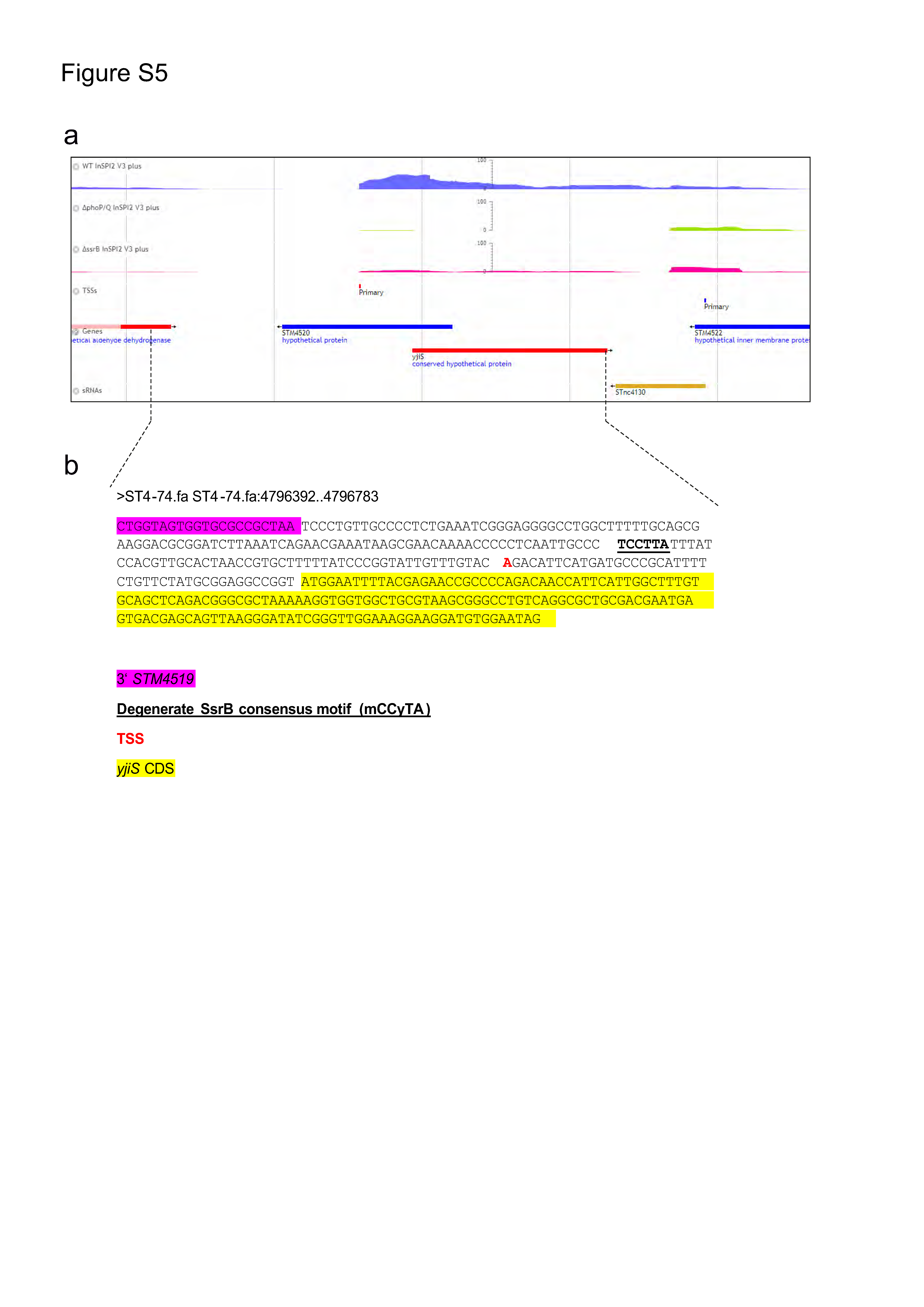

Supplement: uqae026_Supplemental_Files [file uqae026_supplemental_files.zip › Supplementary Figure_5.tif]
